# Basic Psychological Needs Satisfaction: A Way to Enhance Resilience in Traumatic Situations

**DOI:** 10.3390/ijerph19116649

**Published:** 2022-05-29

**Authors:** Maria-Jose Lera, Shadi Abualkibash

**Affiliations:** 1 Department of Psicología Evolutiva y de la Educación, Universidad de Sevilla, 41018 Sevilla, Spain; 2 Department of Psychology, An-Najah National University, Nablus P400, Palestine; shadi.k@najah.edu

**Keywords:** adolescence, basic psychological needs, resilience, trauma

## Abstract

Background/Objective: The impact of traumatic events on resilience and the mediating factors creates specific interest in a conflict context. This study has explored the relationship between the satisfaction of Basic Psychological Needs (BPN) and resilience in adolescents exposed to different levels of adversity in Palestine. Method: A total of 837 eighth-, ninth- and tenth-grade students from the Gaza Strip (*n* = 300) and the West Bank (*n* = 537) completed three questionnaires to assess trauma, BPN satisfaction, and resilience. Results: The results showed a significant difference between the Gaza Strip (0.61) and the West Bank (0.29) in exposure to traumatic events; in both contexts, the BPN satisfaction was associated positively with resilience; in the West Bank the BPN satisfaction mediates the negative impact of trauma on resilience, and in the Gaza Strip, with the higher level of trauma, the BPN satisfaction interacts with trauma, positively affecting resilience. Conclusions: The results highlight the importance of satisfying BPN and indicates the importance of implementing intervention programs designed to satisfy BPN as a way of strengthening resilience in youth people living in traumatic situations

## 1. Introduction

The impact of war and conflict situations on child development was a particularly important issue in the Gaza Strip. Research in the field has shown that experiencing traumatic events, such as large-scale bombings, home demolitions, the death of loved ones and neighbors, among others, and also the ensuing sequelae, such as lacking infrastructure (medical care, traffic, electricity, etc.) is a constant stressor, which is linked to emotional problems, anxiety, and the onset of post-traumatic stress symptoms [1,2], and also behavioral disorders, bed-wetting, and language impairments [3] However, despite the harshness of the attacks, not all of the children in Gaza are equally affected. Research findings reveal that between 20% and 30% of the boys and girls from the Gaza Strip show no symptoms of post-traumatic stress [4,5,6], and a study carried out after Operation Cast Lead in 2009 found that half of the adolescent sample exhibited neither anxiety nor depression [7].

Those children without post-traumatic stress symptomatology have received considerable academic attention in Gaza Strip since the late 1990s, recognizing them as resilient. Resilience indicates the ability to maintain positive psycho-social functioning despite the environmental adversity [8]. These studies reported that the children in Gaza who hold positive attitudes towards life, have a strong social and ideological commitment to their community, and enjoy positive social networks and interpersonal relationships, exhibit high self-esteem, healthy coping mechanisms, and creativity when it comes to problem solving [9].

An examination of the features of their families revealed that these positive attitudes toward life and adaptation (affiliation and social engagement, and successful coping strategies) were not related to the severity of the trauma but to their immediate surroundings; this means that children from families that practice non-punitive parenting, who promote good mental health as well as secure attachment styles, exhibited these positive attitudes. On the other hand, those children that experience insecure-avoidant attachment, and with mothers who take the punitive route, experienced trauma in an overall negative light [10]. From this perspective, the emphasis is placed on defining those aspects that facilitate optimal and adaptive development, despite adversity. The results indicate that a family that provides unconditional support, building secure emotional bonds in a supportive, committed community, increases the potential of overcoming the impact of trauma.

New research in Gaza has started to use a questionnaire designed to measure resilience and which covers three factorial dimensions: contextual, individual, and relational [11], instead of assessing resilience as the non-presence of clinical conditions (depression and anxiety) or post-traumatic stress disorder (PTSD). Those more recent studies have shown a negative effect of adversity on resilience and post-traumatic growth [5]; that means that adverse situations directly affect daily life, bringing about stress and anxiety that manifest in a set of post-traumatic stress symptoms among most of the population.

Taking into consideration the situation of the Gaza Strip before and after 2008, it could shed some light on explaining these findings. The Gaza Strip is internationally known for the hardship that its inhabitants experience daily, although during the 2009 bombing campaign (Operation Cast Lead), the intensity of the attacks far exceeded any strikes before this date. The researchers could argue that the severity of adversity directly affects resilience and the ability to overcome it.

Additionally, there is a continuing interest in exploring the processes that may mediate between experiencing traumatic events and resilience [12]. In this sense, the self-determination theory has addressed the link between motivation and its impact on performance and well-being, focusing on exploring what exactly enables intrinsic motivation and leads to involvement in activity and commitment. Self-determination theory is based on the tenet that the fulfilment of three Basic Psychological Needs (BPN) including autonomy, competence, and relatedness, is essential for positive functioning and when these basic psychological needs are fulfilled, optimal psychological well-being should occur [13]. Ryan and Deci [14] argued that satisfaction of the BPN for autonomy, competence, and relatedness improves well-being, and strengthens inner resources related to resilience, whereas frustration in these three areas increased vulnerability to defense mechanisms and psychopathology [15,16]. Deci and Ryan proposed that psychological need satisfaction acts as a buffer in times of stress, reducing both initial appraisals of stress, and encouraging adaptive coping after stress-related events occur [13].

The need for autonomy represents individuals’ inherent desire to feel volitional and to experience a sense of choice and psychological freedom when carrying out an activity [15,16]. The need for competence is defined as individuals’ inherent desire to feel effective in interacting with the environment [14,17]. The need for relatedness is defined as individuals’ inherent propensity to feel connected to others, that is, to be a member of a group, to love and care, and be loved and cared for [18].

The findings suggest that paving the way for contextual conditions, which allow humans to feel a much-needed sense of autonomy, competence, and relatedness are conducive to increased satisfaction and prosperity, as well as co-benefits associated with greater efficacy [19]. Researchers examining self-determination theory have found consistent results; the social environments that support the satisfaction of all three basic psychological needs facilitate autonomous motivation, psychological and physical well-being, and performance [14].

Some authors argue that satisfying BPN would predict higher levels of resilience and the best use of resources, whereas BPN frustration would be associated with depression and dissatisfaction with life [20].

A study carried out in four countries covering different cultures observed a positive BPN satisfaction relationship between vitality and life satisfaction and found BPN frustration to be linked to depressive symptoms. The conclusion drawn is that BPN satisfaction provides essential nutrients for optimal human performance, as shown across different cultures [21].

Therefore, we understand resilience as a shared human ability that emerges when faced with adverse situations and when certain characteristics are met. It would be valuable to conduct research in other regions that experience adversity to a different degree, using the same unit of measure to compare the results. Additionally, testing the role of BPN satisfaction in relation to resilience would provide more relevant information in order to design psychological interventions.

Thus, we focus on two different contexts with varying degrees of adversity: the Gaza Strip and the West Bank Although both territories are under occupation their inhabitants face different types of adversity. The Gaza Strip is totally isolated and periodically suffers intense attacks (bombs), also most of their population are refugees. The West Bank is surrounded by a wall, and its population face direct problems regarding lack of mobility and daily attacks by armor munition. We hypothesize that:

(1) Trauma negatively affects resilience; (2) BPN satisfaction operates as a mediating factor from trauma to resilience in both samples; and (3) there is an interaction between BPN satisfaction and exposure to traumatic situations, which in turn predicts resilience in both samples.

## 2. Materials and Methods

### 2.1. Participants

A total of 837 Palestinian adolescents participated in the study; 300 from the Gaza Strip (150 boys and 150 girls, aged 14 years (SD = 0.95)), and 537 from the West Bank (242 boys and 294 girls, aged 13.5 years (SD = 0.5)).

The Gaza Strip data were collected from ten UNRWA schools (United Nations Relief and Works Agency for Palestine Refugees in the Near East) across its five governorates (North Gaza: Jabalia; Beit Lahia; Beit Hanoun. Gaza: Deir al-Balah; Khan Younis; and Rafah).

Two schools from each governorate were randomly selected. Eighth-, ninth- and tenth-grade students (all students gave their informed consent for inclusion before they participated in the study) were asked to return a signed parental consent form to be able to participate in the study. Of those who gave their permission, 30 students per school were chosen at random. The selected participants in each school were invited to one of the classrooms and completed the questionnaires under the supervision of the school counsellors trained on the use of these scales. All participants were from refugee families living in refugee camps in Gaza. Data collection took place in December 2016.

In the West Bank, 25 randomly selected public schools across all eight northern Palestinian governorates (Jenin, Nablus, Nablus South, Qalqilya, Tubas, Quelaltya Jericho, Salfit, Tulkarm) took part in the study. Ten students were randomly chosen from each eighth- and ninth-grade class after the students were asked to return a signed parental consent form to be able to participate. Like their Gaza counterparts, they completed the questionnaires with the support of a counsellor. Students were from low socioeconomic background families living in the northern areas of the West Bank. Data collection took place in the spring of 2015. The study was conducted in accordance with the Declaration of Helsinki, and the protocol was approved by the Ethics Committee of University of Seville, Spain.

### 2.2. Instruments

The participants completed three questionnaires:

Traumatic experiences: The Checklist of Traumatic Experiences CTE [22] is a 34-item questionnaire, which covers events that are commonplace in Palestine, such as being arrested, injured, a witness to shelling, and having one’s home demolished. The adolescents reported whether they were exposed to these events (Yes = 1; No = 0). For research purposes, we generated an index with a range between 0–1, with 0 indicating not having suffered any of the traumatic situations, and 1 indicating that all of the traumatic events presented were experienced. The CTE was previously developed, evaluated to ensure its validity and reliability, and adapted into Palestinian Arabic in the Gaza Strip [22];

The Basic Psychological Needs Satisfaction scale (BPNS) [14], featuring 21 items that assess levels of satisfaction in terms of autonomy (seven items), competence (six items), and relatedness (eight items). On a scale of 1 to 7, participants rated how well the descriptions matched their perceptions and feelings (1 = Definitely not true, 7 = Definitely true) (for example, relatedness: “People in my life care about me”; competence: “In my life I do not get much of a chance to show how capable I am”; and autonomy: “In my daily life, I frequently have to do what I am told”). In this study, the total mean would be taken as the general satisfaction level for all three BPN dimensions. The BPN scale was previously adapted and translated into Palestinian Arabic by the researchers, Diseth, Danielsen, and Samdal (2012) [23].

Resilience. The third questionnaire, the 28-item Child and Youth Resilience Measure (CYRM-28) [24], is the most frequently used tool for measuring resilience among these groups. It assesses resources and strengths, broken down into three dimensions: context (educational, cultural, spiritual); individual (social skills, peer support, personal skills; and caregiver (physical and psychological) (for example: “I feel supported by my friends”, “I am aware of my own strengths”, and “I am proud of my culture/ethnic background”). On a scale of 1 to 5, participants assessed to what extent this situation encompasses their own experience. The CYRM-28 was previously adapted and translated into Palestinian Arabic in the Gaza Strip [6]. A high score is viewed as extremely resilient. We analyzed all three dimensions separately (values from 1 to 5).

### 2.3. Data Analysis

Regarding the first hypothesis, to test the relationships between psychological resilience, psychological basic needs satisfaction (BPNS), and trauma, we used descriptive data, Pearson correlations, and ANOVA test -to compare the differences between the two samples, using the SPSS (version 21, IBM, An-Najah National University, Nablus, Palestine).

To assess hypotheses two and three, we used the structural equation modelling [25] using the AMOS (Analysis of Moment Structures) software (SPSS version 21).

**Figure 1:**
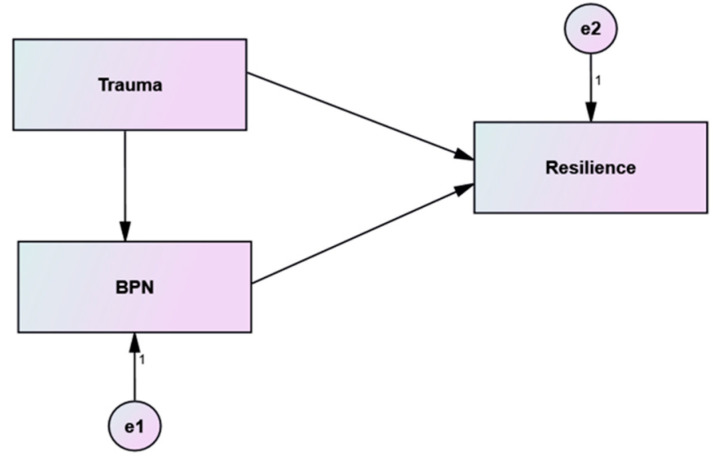
Hypothesized Structural Model (BPNS medidates the effect of trauma on resilience).

Two models were proposed: the first model hypothesizes that BPN satisfaction is a mediating factor from trauma to resilience for both groups (the mediation model), as shown in Figure 1.

**Figure 2:**
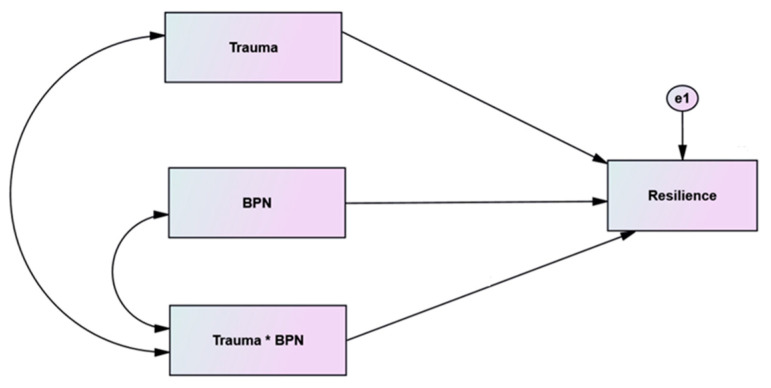
Hypothesized Structural Model (the effect of interaction of trauma and BPNS on resilience).

The second model hypothesizes that BPN satisfaction, exposure to traumatic situations, and the interaction between both (a product of them) predicts resilience [26], as shown in Figure 2.

## 3. Results

**H1:**  *Descriptive analysis and correlations: trauma, BPNS, and resilience*.

The descriptive analysis for both samples can be seen in Table 1.

To evaluate whether individuals from the Gaza Strip showed a higher level of trauma exposure than those from the West Bank, we used a comparison of means (ANOVA). Data revealed that the Gaza Strip sample presented the highest values of exposure to traumatic situations, F (1, 836) = 660.37, *p* < 0.001, η2 = 0.442); the mean for the West Bank was 0.29 and for Gaza Strip 0.61, on a scale of 0 to 1.

The comparison of means for BPN satisfaction indicates that the Gaza Strip sample scored lower on this scale, F(1, 836) = 92.17, *p* < 0.001, η2 = 0.220), and had a greater mean score on resilience compared with the West Bank sample, F(1, 836) = 69.31, *p* < 0.001, η2= 0.077 (see Table 1).

To evaluate the first hypothesis, we explored whether there were any resilience dimensions significantly associated with trauma under both situations of adversity. The Pearson’s correlation index showed the contextual dimension of resilience was negatively related to traumatic events in the Gaza Strip sample (r(300) = −0.16, *p* < 0.01) and in the West Bank sample too (r(537) = −0.11, *p* < 0.01), demonstrating a similar negative impact of traumatic events on the contextual resilience for both contexts. However, resilience as a general index was not associated with traumatic events in Gaza. Therefore, the results confirmed partially the first hypothesis, as trauma was negatively associated only with a dimension of resilience.

**H2:**  *BPNS as a mediator*.

**Figure 3:**
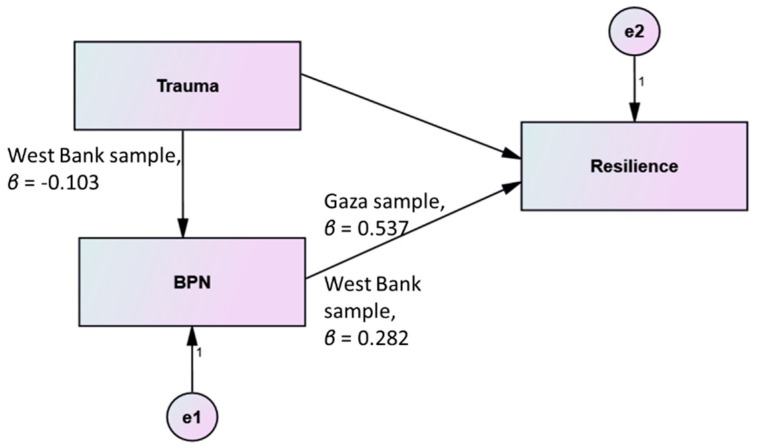
Structural Model BPNS mediates the effect of trauma on resilience.

In response to the second hypothesis, that is, whether BPN satisfaction can serve as a mediator of resilience (see Figure 3), we conducted a SEM data analysis related to multiple-group analysis for both models, using the AMOS multiple-group analysis; standardized regression weights were estimated for both groups separately; 537 adolescents from the West Bank and 300 adolescents from Gaza Strip. The results are presented in Table 2.

Two insignificant paths should be eliminated from the Gaza Strip model: the path from trauma to BPNS and the path from trauma to resilience, which indicates that the mediating effect of BPNS between trauma and resilience does not exist in the Gaza Strip model. On the other hand, one insignificant path should be eliminated from the West Bank model: the path from trauma to resilience, which indicates that there is an indirect effect between trauma and resilience through BPNS in the West Bank model.

The West Bank model could prove a stable model after deleting the path from trauma to resilience. It is also a recursive model, which is confirmed because of the one-way relation between variables. The minimum discrepancy, that is, the CMIN value from the model fits analysis was 2.635; the DF of 1 translated into a CMIN/DF of 2.635; and the RMSEA estimate of 0.055 successfully supported the model. Bentler’s CFI for the present model was 0.969, meaning that the proposed model fits the data according to this index. For GFI, AGFI, and NFI, the values were 0.997, 0.981, and 0.952, respectively; thus, the proposed model fits the data very well.

The standardized path coefficient from trauma to BPNS was negative and significant (β = −0.103, *p* = 0.016), and from BPNS to resilience it was positive and significant (β = 0.298, *p* < 0.001).

**H3:**  *BPNS interactive effects on resilience.*

To test the third hypothesis, we set up a second model that hypothesized that BPN satisfaction, exposure to traumatic situations, and the interaction between BPN satisfaction and exposure to traumatic situations (a product of them) predict resilience (Hawkley et al.), as shown in Figure 2; the standardized regression weights were estimated for both groups separately. The results are presented in Table 3.

As shown in Table 3, one insignificant covariance should be eliminated from the Gaza Strip model, as the correlation coefficient between trauma and BPNS was insignificant. On the other hand, two insignificant paths should be eliminated from the West Bank model: the path from trauma to resilience and the path from interaction to resilience, which indicate that only BPNS affects resilience in the West Bank model.

The Gaza Strip model could prove to be a testable model after deleting the covariance between trauma and BPNS. It is also a recursive model, which is confirmed because of the one-way relation between variables. The minimum discrepancy, that is, the CMIN value from the model fits analysis was 1.182; the DF of 1 translated into a CMIN/DF of 1.182; and the RMSEA estimate of 0.025 successfully supported the model. Bentler’s CFI for the present model was 1.00, meaning that the proposed model perfectly fits the data according to this index. For GFI, AGFI, and NFI, the values were 0.998, 0.980, and 0.999 respectively; thus, the proposed model fits the data very well.

The standardized path coefficient from trauma to resilience was positive and significant (β = 0.799, *p* = 0.038); from BPNS to resilience it was positive and significant (β = 0.845, *p* < 0.001); and from interaction to resilience, it was positive and significant (β = 0.900, *p* = 0.028).

**Figure 4:**
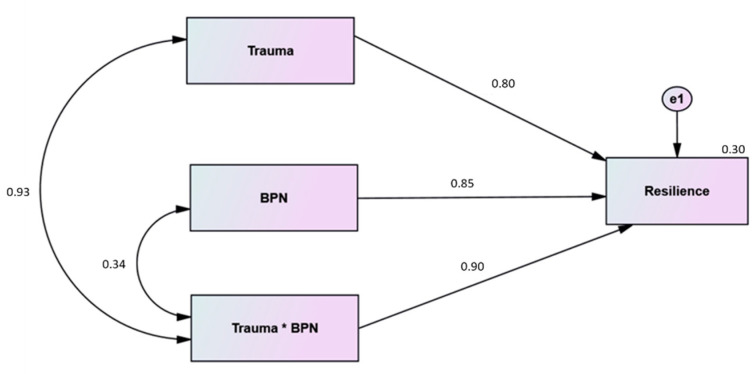
Modified Structural Model for Gaza Strip (effect of interaction of trauma and BPNS on resilience).

The R2 for resilience was moderate (0.302), which in turn indicates that BPNS, trauma, and the interaction between both play a modest role in explaining the variance of resilience in Gaza Strip. Therefore, the third hypothesis was partially supported in the Gaza Strip; however, it was not supported in the West Bank. Figure 4 shows the Gaza Strip model.

## 4. Discussion

The researchers explored the relationship between trauma, BPN satisfaction, and resilience; the first analysis led us to the conclusion that adolescents in the Gaza Strip are subject to greater adversity, presenting high values of traumatic event experience compared to the West Bank sample. However, despite this high trauma exposure, the Gaza Strip sample showed a very high resilience mean score; these results are backed by other studies that highlight Gaza Strip adolescents’ enhanced ability to confront adversity [7] (Aitcheson et al., 2017).

Therefore, the assumption that a high degree of adversity would more negatively impact on resilience was not confirmed. The Gaza Strip data did not reveal a significant correlation between adversity and total resilience; however, this relationship was significant in the West Bank sample, yielding a lower adversity rate and lower resilience values, although this correlation was weak. These results do not support the prediction that the greater the trauma severity, the greater its negative impact on resilience.

An analysis of the link between trauma and the different resilience dimensions (individual, relational, and contextual) did show a negative yet significant and small relationship between trauma and the contextual resilience dimension in both the Gaza Strip and the West Bank. Data indicate that trauma, be it of lesser or greater adversity, partially and significantly affects resilience negatively, particularly in the contextual dimension. This dimension covers aspects related to community values, such as enjoying community traditions, feeling proud of one’s citizenship, participating in religious and educational activities, and a feeling of belonging to the school, which were associated with less PSTD in the case of trauma. The results indicate that these contextual conditions are more affected by traumatic events of wars than other dimensions of resilience, such as individual characteristics (I am able to solve problems) and relations (I enjoy my family caregivers).

These results only partially support those found in other research studies conducted among Gaza Strip adolescents, which reported that all of the dimensions of resilience are equally affected [5,6](Murad and Thabet, 2017; Thabet and Thabet, 2015). To clarify which dimension of resilience is more affected by trauma, further research should focus on whether the frequency, repetition, and the type of traumatic situations, that Palestinian adolescents are facing in Gaza and the West Bank, are associated with the different impacts on resilience, and which intervention should be provided to minimize it.

Regarding the relationship between BPN satisfaction and resilience, the data revealed a high and positive correlation, showing that BPN satisfaction is associated with resilience, regardless of the adversity level. The Gaza Strip data report a strong correlation (r = 0.54) and the West Bank data report a moderate one, (r = 0.28), indicating that BPN satisfaction can potentially serve as an intervention pathway that not only facilitates resilience, but also, as seen in other research, increases the chances of positive post-traumatic recovery [5]. These results coincide with other studies that confirm a link between BPN satisfaction and resilience in the West Bank [27], as well as other health indicators including more life satisfaction [23], greater psychological well-being [28,29], and more vitality [18]. The contribution made by this study is that there is a positive correlation between BPN satisfaction and resilience in both high- and moderate-adversity environments.

To test the second hypothesis, that is, whether BPN satisfaction operates as a mediating factor, we explored the effect that trauma has on resilience. We observed a mediating effect in the West Bank sample, which means that when trauma negatively correlates with resilience, BPN satisfaction can mediate and reduce the negative impact.

When testing the third hypothesis, the interaction effect of trauma on BNPS was observed in the context of the Gaza Strip, confirming the hypothesis, and showing that trauma, BPN satisfaction, and their interaction predicted 30% of the variance of resilience. The interactive effect between trauma and BPN satisfaction means that the positive effect on resilience multiplies, as trauma and/or BPNS increase.

Therefore, a context that satisfies BPN in adolescents, living under high adversity conditions, will help pave the way for resilience. These results enable us to conclude that BPN satisfaction plays a crucial role in enhancing resilience: it has a mediating effect in the West Bank sample; and it interacts with trauma and predicts even higher resilience in the case of the Gaza Strip, strengthening resilience.

Notably, BPN satisfaction was lower in the Gaza Strip than in the West Bank. Therefore, we need to address the interaction effect with high-level trauma when BPN are very well satisfied, which was not possible in this study.

A limitation of this study has to do with how the variables were measured; all three variables were explored using self-report questionnaires to assess the adolescents’ perceptions, including no other external measure which would lend more validity to the current research. When it came to evaluating trauma, the approach used was to indicate whether the person had experienced the situation in question or not, whereby greater trauma was computed as having gone through more traumatic situations. This measure, however, does not cover repeated adversity, which could have an impact on the ability to recover. We know that traumatic situations experienced in Palestine are repetitive in nature, such as the demolition of homes, which occurs frequently and within the same family, neighborhood, or refugee camp; also, the lack of infrastructure (medical care, traffic, energy) plays a key role in recovery. For further studies it would be useful to consider not only whether someone went through this particular situation but also how many times, and which coping strategies were used, and to carry out longitudinal studies. Therefore, exploring and introducing other variables are needed as contextual variables, such as the provision of demographic data, and some authors suggest that self-efficacy beliefs could make for a possible precursor of resilience [7].

Considering these limitations, these findings can be interpreted as scientific support for claims that the traumatic events that unfold in Palestine hurt adolescents’ resilience, affecting contextual resilience factors. Furthermore, BPN satisfaction can mediate the negative impact of trauma, and in the case of high adversity as in Gaza, BPN satisfaction interacts with trauma, acting additionally as a precursor of resilience and diminishing the negative impact of the trauma.

This study contributes toward underlining the importance of psycho-educational interventions that facilitate BPN satisfaction as a way of building resilience. School-based group interventions are seen as an option for increasing BPN satisfaction in childhood and adolescence, and therefore represent a way of helping to increase the likelihood of achieving optimal development despite adversity.

From this perspective, previous experiences in the West Bank and the Gaza Strip have led to highly satisfactory results after having implemented the Golden5 psycho-educational program, aimed at both teachers and learners; this program is based on the teacher’s role in satisfying BPN and in supporting high self-efficacy beliefs, acting as a driving force behind the student’s psychological well-being [30]. The program proposes a number of strategies organized around five areas that were associated with engagement, well-being, and positive outcomes, such as encouraging positive and warm feedback, suggesting tasks based on students’ interests, and promoting close teacher–student relationships (the program can be found at www.golden5.org. accessed on 10 May 2022).

## 5. Conclusions

The study’s main contribution lies in that BPN satisfaction plays a mediating effect (the West Bank sample) on trauma, and an interactive effect (the Gaza sample) with trauma, limiting its negative impact on resilience. Therefore, a context that satisfies BPN under traumatic conditions will help pave the way for resilience, at least in Palestinian contexts

New research is needed to explore how the different types of trauma appreciated in the West Bank and Gaza have different effects on resilience, and also whether psychoeducational interventions aimed at enhancing BPN satisfaction and self-efficacy beliefs can succeed in mediating and/or interacting with the negative effects of trauma on resilience when facing diverse levels of repeated adversity.

This study may be of interest to those designing and implementing social and educational policies geared toward facilitating resilience in adolescents who find themselves living in environments where traumatic events take place.

## Figures and Tables

**Table 1:** Pearson’s correlations, maximum and minimum scores, number of items, mean, standard deviation, and Cronbach’s alpha for Trauma, BPNS, and Resilience for Gaza and the West Bank.

	Gaza	W. Bank	Gaza	W. Bank	Gaza	W. Bank	Gaza	W. Bank	Gaza	W. Bank	Gaza	W. Bank
	1		2		3		4		5		6	
1. Trauma	-											
2. BPNS	−0.063	−0.103 *	-									
3. Resilience	−0.074	−0.096 *	0.540 **	0.289 **	-							
4. Resil. individual	−0.028	−0.092 *	0.514 **	0.347 **	0.874 **	0.877 **	-					
5. Resil. relational	0.004	−0.056	0.394 **	0.251 **	0.758 **	0.914 **	0.496 **	0.700 **	-			
6. Resil. contextual	−0.160 **	−0.117 **	0.399 **	0.205 **	0.814 **	0.920 **	0.566 **	0.731 **	0.443 **	0.751 **		
Maximum score	1		7		5		5		5		5	
Minimum score	0		1		1		1		1		1	
Number of items	34		21		28		11		7		10	
Mean	0.61	0.28	4.46	5.15	4.15	3.80	4.01	3.47	4.17	4	4.28	3.90
Standard deviation	0.18	0.17	0.51	0.68	0.43	0.67	0.53	0.62	0.60	0.81	0.47	0.79
Cronbach’s alpha	0.9	0.9	0.65	0.77	0.83	0.97	0.72	0.8	0.63	0.78	0.65	0.83

* The correlation is significant at the 0.05 level and ** at the 0.01 level.

**Table 2:** Standardized regression weights for the mediation model (Vaillant).

Gaza			West Bank		
Parameter Description	Standardized Coefficient	*p*-Value	Parameter Description	Standardized Coefficient	*p*-Value
BPN from Trauma	−0.063	0.276	BPN from Trauma	−0.103 *	0.016
Resilience from Trauma	−0.040	0.413	Resilience from Trauma	−0.067	0.104
Resilience from BPN	0.537 **	0.000	Resilience from BPN	0.282 **	0.000

* significant at the 0.05 level and ** at the 0.01 level.

**Table 3:** Standardized regression weights and correlation coefficients for the interaction model (second model).

Gaza			West Bank		
Parameter Description	Standardized Coefficient/Correlation	*p*-Value	Parameter Description	Standardized Coefficient/Correlation	*p*-Value
Resilience from Trauma	0.798 *	0.038	Resilience from Trauma	−0.141	0.645
Resilience from BPN	0.844 **	0.000	Resilience from BPN	0.265 **	0.000
Resilience from Interaction	0.880 **	0.028	Resilience from Interaction	0.075	0.808
Trauma with Interaction	0.930 **	0.000	Trauma with Interaction	0.965 **	0.000
BPN with Interaction	0.289 **	0.000	BPN with Interaction	0.123 **	0.005
Trauma with BPN	−0.063	0.278	Trauma with BPN	−0.103 **	0.018

* significant at the 0.05 level and ** at the 0.01 level.

## Data Availability

The authors confirm that the data supporting the findings of this study are available within the article Supplementary Materials.

## Supplementary Materials

The following supporting information can be downloaded at: https://www.mdpi.com/article/10.3390/ijerph19116649/s1.
